# Influence of Inter-Implant Distance on the Marginal Fit of Milled Structures and Virtual Model Trueness: *In Vitro* Study

**DOI:** 10.1590/0103-644020256422

**Published:** 2025-11-21

**Authors:** Luísa de Lanna Reis Rocha, Ana Carolina Candelas Peixoto, Fábio Henrique de Paulo Costa Santos, Carlos José Soares, Gustavo Mendonça, Flávio Domingues das Neves, Karla Zancopé

**Affiliations:** 1 Department of Occlusion, Fixed Prosthesis and Dental Materials, School of Dentistry, Federal University of Uberlândia, Uberlândia, MG, Brazil.; 2 Department of Dentistry and Dental Materials, School of Dentistry, Federal University of Uberlândia, Uberlândia, MG, Brazil; 3 Department of General Practice, Virginia Commonwealth University, School of Dentistry, Richmond, Virginia, United States

**Keywords:** accuracy, dental implant, digital impression, digital intraoral scanning, prosthesis, computer-aided design

## Abstract

Este estudo in vitro avaliou o impacto de três distâncias entre implantes (7,14 e21 mm) na veracidade e distorção superficial de modelos gerados por meio de escaneamento intraorais, bem como a desadaptação de estruturas fresadas, analisada por microscopia eletrônica de varredura (MEV). A desadaptação na interface entre as estruturas e os intermediários protéticos também foi mensurada por meio de radiografia digital e MEV. Três modelos com diferentes distâncias entre implantes foram escaneados (n = 10), e um escaneamento de referência para cada modelo foi obtido com scanner de bancada. A veracidade foi avaliada por meio de comparações tridimensionais (3D) das imagens. Cinco estruturas em cobalto-cromo foram fresadas para cada grupo, e as desadaptações nas faces mesial e distal de ambos os implantes foram avaliadas por MEV e radiografia digital. A análise estatística foi realizada com α = 0,05. Não foram encontradas diferenças significativas na veracidade 3D entre as diferentes distâncias entre os implantes (p = 0,373), nem nos valores de desadaptação mesial (p = 0,075) e distal (p = 0,720) do implante anterior. Da mesma forma, não houve diferenças significativas nos valores de desadaptação mesial (p = 0,103) ou distal (p = 0,426) do implante mais posterior. Foi identificada uma forte correlação entre as análises de desadaptação realizadas por radiografia e MEV (r de Pearson = 0,819; p = 0,045). A distância entre os implantes não influenciou significativamente a veracidade ou a adaptação das estruturas fresadas. Todas as estruturas apresentaram boa adaptação aos intermediários protéticos. A forte correlação entre as análises radiográficas e por MEV sugere que a radiografia digital é uma ferramenta confiável para detectar desadaptações entre estruturas fresadas e implantes.

## Abstract (en)

This *in vitro* study evaluated the impact of three inter-implant distances (7, 14, and 21 mm) on the trueness and surface distortion of models generated using intraoral scan bodies, as well as the misfit of milled structures assessed through scanning electron microscopy (SEM). Misfit at the multi-unit structure interface was also measured using digital radiography and SEM. Three typodont models with varying inter-implant distances were scanned (n = 10), and a reference scan was obtained using a desktop scanner. Trueness was evaluated through three-dimensional (3D) image comparisons. Five cobalt-chromium structures were milled for each group, and misfits at the mesial and distal faces of both implants were assessed using SEM and digital radiography. Statistical analysis was performed with α = 0.05. No significant differences were found in 3D trueness across the inter-implant distances (*p = 0.373*) or in mesial (*p = 0.075*) and distal (*p = 0.720)* misfit values of the most anterior implant. Similarly, no significant differences were found for mesial (*p = 0.103*) or distal (*p = 0.426*) misfit values of the most posterior implant. A strong correlation was identified between X-ray and SEM analyses of misfit (Pearson’s r = 0.819, *p = 0.045*). Inter-implant distance did not significantly influence the trueness or fit of the milled structures. All milled structures adapted well to the implant abutments. The strong correlation between X-ray and SEM analyses suggests that digital radiography is a reliable tool for detecting misfit between milled structures and implants.

**Figure:**
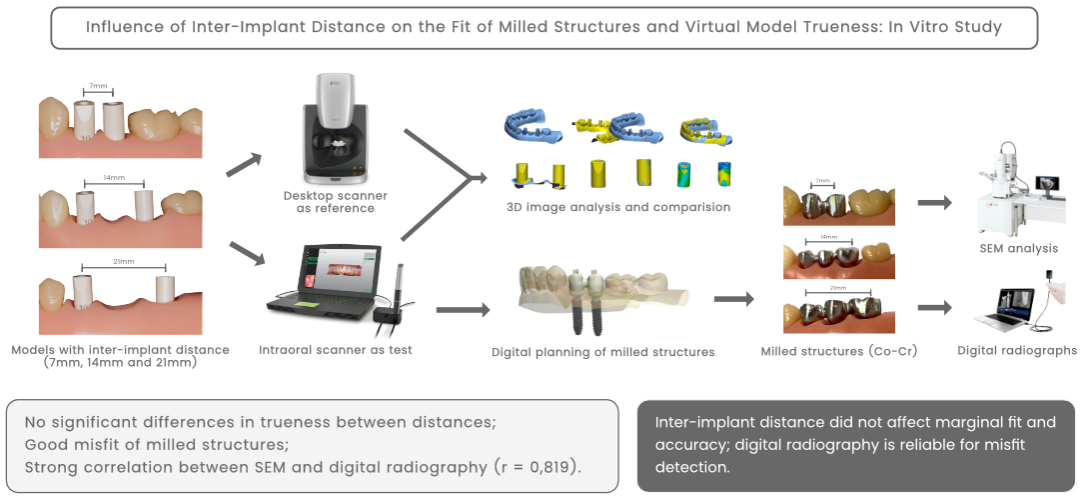

## Introduction

Intraoral scanning plays an essential role in implant dentistry by enabling the precise digital fabrication of implant-supported restorations. ^1^ ^,^ ^2^ This method must accurately replicate three-dimensional (3D) representations of implants and prosthetic components to facilitate successful digital planning for implant-based prostheses. ^1^ Accurate scanning is critical for the success of prosthetic rehabilitation and the production of well-fitted indirect restorations. ^3^ ^,^ ^4^ ^,^ ^5^ ^,^ ^6^ ^,^ ^7^ The precise positioning and inclination of the intraoral scan body (ISB) are essential for the accurate transfer of implant data. ^2^ ^,^ ^8^ ^,^ ^9^ ^,^ ^10^ ^,^ ^11^

A passive fit of prostheses is crucial to prevent stress on implants. ^8^ Continuous uneven forces exceeding physiological limits can lead to biomechanical problems within the implant system, causing component fatigue and bone strain. These issues may result in both prosthetic and biological complications, such as loosening of the restoration, peri-implant bone loss, and premature failure of implant components. ^7^ ^,^ ^12^ Improving the marginal adaptation of milled structures requires identifying factors that influence the acquisition stages. ^3^

While previous studies have primarily examined the accuracy of intraoral scanners in implant dentistry ^2^ ^,^ ^5^ ^,^ ^6^ ^,^ ^7^ ^,^ ^8^ ^,^ ^13^ ^,^ ^14^ ^),^ few have explored the impact of inter-implant distances on the marginal fit and accuracy of virtual models. Scanning accuracy can be affected by various factors, including the scanning technology used ^3^ ^,^ ^11^ ^,^ ^15^, clinician experience, scanning technique ^15^, as well as the design and material of the scan bodies and the distance between them. ^1^ ^,^ ^11^ Patient-related factors, such as limited mouth opening, an oversized tongue, and hypersalivation, can also compromise the accuracy of virtual models. ^7^ ^,^ ^15^ Differences between intraoral and desktop scanners, as well as between materials like PEEK and titanium, can lead to variations in data acquisition and model trueness. ^15^ ^,^ ^16^ ^,^ ^22^ The geometry and reflectivity of scan bodies, combined with scanner-specific software algorithms, may affect how accurately implant positions are recorded and translated into virtual models. Thus, understanding the impact of inter-implant distance in isolation, while controlling for other variables, is essential to delineate its actual influence on digital workflows.

Clinical methods, such as tactile exploration^16^ and radiography ^17^, are commonly used to assess the marginal fit of milled structures. However, these methods may lack the precision of laboratory-based techniques, such as scanning electron microscopy (SEM), optical microscopy, and micro-computed tomography (micro-CT), which are capable of detecting misfits with micrometer-level accuracy. ^5^ ^,^ ^12^ Few studies have investigated correlations between clinical and laboratory methods. Establishing these correlations is crucial for bridging the gap between laboratory findings and clinical observations. ^16^

The distance between scan bodies may influence the trueness of virtual models and the fabrication of prosthetic structures. ^2^ ^,^ ^8^ ^,^ ^9^ ^,^ ^10^ ^,^ ^11^ ^,^ ^13^ However, the potential impact of the distance between ISBs at the time of scanning on the marginal adaptation of milled structures obtained through a fully digital workflow remains unclear. Therefore, this study aimed to evaluate the effect of the distance between two implants on the surface distortion of models generated using ISBs, the misfit of milled structures assessed through scanning electron microscopy (SEM), and the comparison of misfit levels at the multi-unit structure interface as observed in digital radiographs and SEM analysis. The null hypothesis was that the distance between ISBs during scanning would not affect the trueness of the model or the marginal fit of the milled structures.

## Materials and methods

**Figure 1:**
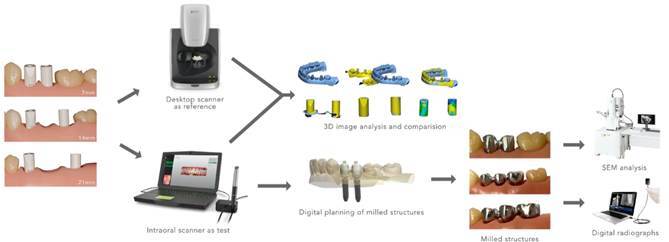
Experimental workflow.

The experimental workflow is depicted in Figure 1. Three partially edentulous typodonts (MOM Manequins Odontológicos, Marília, Brazil) with varying distances between implant analogs were used as reference models. Typodonts were chosen to facilitate implant placement, scanning, and evaluation procedures, providing a standardized and reproducible in vitro environment. Implant-supported prostheses comprising two or three splinted elements screwed onto two implants were selected as treatment options. Two implants (Helix GM 3.5 x 11.5, Neodent, Curitiba, Brazil) were placed in each model, with inter-implant distances of 7, 14, and 21 mm, measured using a digital caliper (Mitutoyo, Tokyo, Japan). The inter-implant distances selected for this study were based on commonly encountered edentulous spans in clinical practice. Specifically, a 7 mm gap simulates the absence of the first and second premolars; 14 mm simulates the absence of both premolars and the first molar; and 21 mm represents a posterior edentulous space comprising the second premolar, first molar, and second molar. These values align with average mesiodistal tooth dimensions reported in anatomical and prosthetic planning studies. ^21^ ^,^ ^23^ The implants were embedded in self-polymerizing acrylic resin (Acrylic Resin Pattern Bright Red, Kota, Aichi, Japan) to ensure stability and prevent movement of the implants during testing. Multi-unit abutments compatible with the implant system (GM MultiUnit 2.5, Neodent, Straumann, Basel, Switzerland) were installed with the manufacturer-recommended torque of 32 N/cm.

This study did not involve human participants, animal subjects, or any biological samples that would require ethical approval. Therefore, approval from an ethics committee was not required, in accordance with the Brazilian Resolution CNS nº 510/2016, which states that research not involving human participants or sensitive data is exempt from ethical review. The methodology employed in this research adheres to all relevant international and national guidelines, including the Organisation for Economic Co-operation and Development (OECD) Guidelines for the Testing of Chemicals. These standards ensure compliance with ethical principles and guarantee the reliability and reproducibility of results in vitro studies.

### Scanning

**Figure 2:**
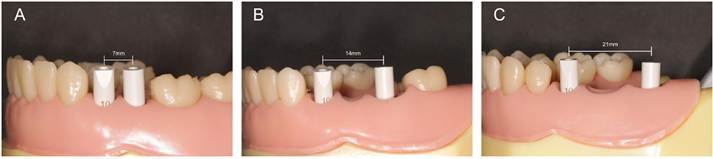
Models with inter-implant distances: A) 7 mm, B) 14 mm, and C) 21 mm.

ISBs compatible with the multi-unit system were installed (Figure 2). Following the findings of a pilot study, the bevel features of the implant scan bodies were intentionally positioned in a misaligned configuration as the preferred setup. Lighting conditions were standardized, and a single operator performed all scans to ensure consistency and repeatability. The scanner was calibrated for each model, and the ISBs remained in the same position until all repeated scans for each cast were completed.

Image acquisition was conducted in two phases. In the first phase, a desktop scanner (inEos X5, Dentsply Sirona, Charlotte, USA) was used as the reference method. For the test groups, scans were performed using an intraoral scanner (Virtuo Vivo, Straumann, Basel, Switzerland, Version 3.10.1.1335), which was selected for its proven precision, compatibility with the scan bodies used, and validated performance in prior studies. Scanning was performed according to the manufacturer's protocol by a single experienced operator.

The models were scanned ten times in each lower quadrant, with variations in inter-implant distances between groups (n = 10). The order of scans was randomly alternated between groups using a true random number generator (Randomness and Integrity Services Ltd. (n.d.). RANDOM.ORG - True Random Number Service). Scanning followed the manufacturer's guidelines, starting in the posterior region of the right quadrant, progressing along the occlusal surface of the arch, and employing zigzag movements in the anterior tooth region. The process continued with scans of the buccal region, followed by the lingual region. The resulting Standard Tessellation Language (STL) files were saved in a digital folder and sent to a dental laboratory (D-Lab Digital Excellence, Curitiba, Brazil).

## Image Analysis

**Figure 3:**
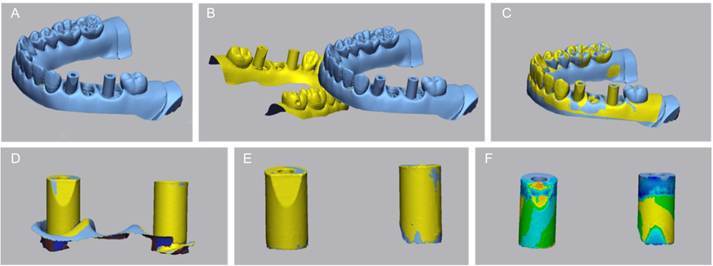
Trueness assessment through comparison between desktop scans and intraoral scans. A: Standard Tessellation Language (STL) file of the reference group imported. B: STL file from the intraoral scan imported. C: Initial alignment performed using the "Initial Alignment" tool. D: Sections approximately 2 mm below the gingival margin were removed from the digital models. E: Modified files aligned using the "Best Fit Alignment" tool. F: Intraoral scan bodies (ISBs) compared in three dimensions (3D) using a color scale threshold of 0.5 μm.

After completing all scans, the STL files were imported into image analysis software (Geomagic Control X, 3D Systems, Version 2018.1.1) to compare the test group (intraoral scans) with the reference group (desktop scans) under mesh overlap conditions. The analysis steps are outlined in Figure 3.

The STL file generated by the desktop scan (reference group) was first imported into the software and set as the reference layer. Subsequently, an STL file from the intraoral scan, corresponding to the same inter-implant distance, was imported. An initial alignment of the two models was performed using the "Initial Alignment" tool. To improve measurement accuracy, sections approximately 2 mm below the gingival margin were removed from the digital models. The modified files were then aligned using the "Best Fit Alignment" tool, with the areas surrounding the ISBs clipped to minimize potential discrepancies. Finally, the ISBs were compared in 3D using a color scale threshold of 0.5 μm. ^20^ Average (AVG) data were extracted and organized into tables in Microsoft Excel for further analysis. An experienced software operator carried out all procedures.

### Manufacturing of Structures

**Figure 4:**
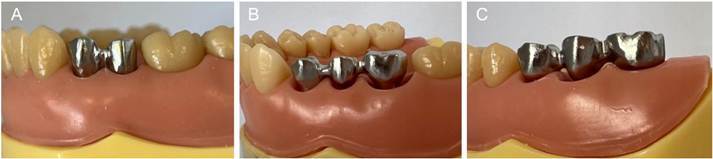
Milled structures screwed onto the implant multi-unit abutments in models with inter-implant distances: A. 7 mm, B. 14 mm, and C. 21 mm.

The arch files in STL format were exported to a private laboratory (DLab Digital Excellence, Curitiba, Brazil) for the manufacturing of structures (five specimens per group). A random integer generator (Random.ORG) was used to randomly select five files from the ten intraoral scans performed for each group. Cobalt-chromium structures (Magnum Splendidum, MESA, Brescia, Italy) were digitally planned and manufactured using a milling machine (Ultrasonic 20 Linear, DMG MORI, Tokyo, Japan). The fabricated structures were then secured to the abutments (Figure 4) with a torque of 10 N/cm, as recommended by the manufacturer.

### SEM Analysis

SEM analysis (Zeiss EVO MA10, Oberkochen, Germany) was conducted by an experienced, blinded professional (L.A.J.S.). Each cobalt-chromium metal structure was positioned on its corresponding model, and the misfit was assessed by measuring the vertical and horizontal gaps between the structure platform and the multi-unit analogs at the mesial and distal faces of the interface. Measurements were taken at 500x magnification with an extra high tension of 10.00 kV. To standardize the inclination, all structures were mounted on a device within the SEM that ensured consistent angulation and positioning.

For each misfit measurement, four images were captured per model: one mesial and one distal image for each implant. This approach enabled the identification of misfits at the junction of the multi-unit structure. The mean misfit measurements for the mesial and distal faces of each implant were calculated and compared across different inter-implant distances. For reference, the acceptable misfit for gold alloy structures is typically less than 70 μm ^19^, a threshold used as a benchmark in this study.

### Radiographic Analysis

Standardized digital periapical radiographs were obtained for all structures screwed onto the multi-unit abutments. A portable digital X-ray device (Diox, Micro Image, London, United Kingdom) was used, following the manufacturer's instructions. The device was positioned perpendicular to the digital sensor, which was placed behind the structures, to ensure accurate imaging. The X-ray exposure time was set to 0.30 s, and all radiographs were taken under identical conditions and positioning for consistency. Misfit detection in the radiographs was conducted by a blinded observer (K.Z.) who was not involved in the research. Measurements of the visible misfits were performed using ImageJ (ImageJ 1.x) software following the same reference points used in SEM analysis. This approach allowed for quantitative comparison between the two methods.

### Statistical analysis

The Shapiro-Wilk test was used to assess the normality of the data, while Levene’s test evaluated the homogeneity of variances, ensuring that the assumptions were met before conducting further statistical analyses. Analysis of variance (ANOVA) was performed to compare the 3D comparison data between the test and reference groups, as well as the misfit measurements in micrometers (μm) obtained from SEM images. The Spearman correlation coefficient was used to determine associations between distances measured through 3D comparison and those measured microscopically by SEM. Furthermore, Pearson's correlation coefficient was calculated to assess the relationship between the misfit values obtained from SEM and the radiographs of the same samples. Statistical significance was set at α = 0.05.

## Results

### Image analysis

**Figure 5:**
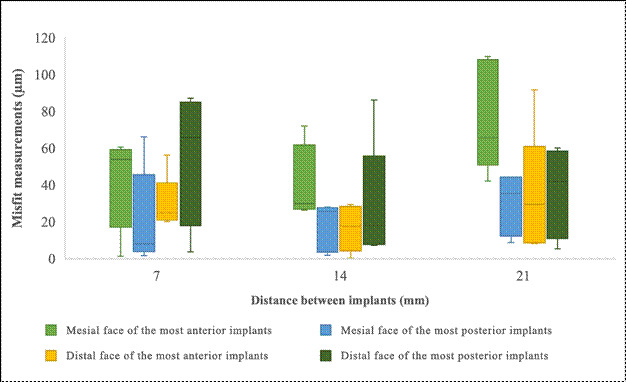
Discrepancy and misfit measurements across different inter-implant distances. Left: Boxplot showing discrepancies between the virtual model and the reference scans. Right: Boxplot showing misfit measurements at the mesial and distal faces of the most anterior and most posterior implants.

The 3D comparisons of models with implants placed at distances of 7 mm, 14 mm, and 21 mm revealed no significant differences in mean discrepancies (μm) (*p = 0.373*), as illustrated in Figure 5. Across all inter-implant distances, the mean discrepancy between the virtual models and the reference scans remained relatively constant. The trueness of the virtual models showed no significant variation across the tested distances, with discrepancies ranging from 0.02 mm to 0.04 mm. These findings indicate that the accuracy of the generated virtual models was consistent and acceptable, regardless of the distance between implants.

### SEM analysis

No vertical misfits were observed; all detected misfits were horizontal*.* Descriptive statistics for the misfit values revealed a mean of 42.0 ± 27.9, indicating high variability. The median value was 32.5, indicating slight right skewness, which is further supported by a positive skewness value of 0.626. No significant differences were observed in the misfit measurements of the mesial faces of the most anterior implants (*p = 0.075)*; however, the effect size was large, as shown in Figure 5. Similarly, comparisons of distal measurements revealed no significant differences in mean misfits (*p = 0.720)*, with a tiny effect size. For the mesial misfit measurements of the milled structures of the most posterior implants, no significant differences in mean misfits were detected (*p = 0.103),* although the effect size was large. In the distal measurements of the same implant, no significant differences were found (*p = 0.426*), with an effect size that was small.

### Radiographic analysis

**Figure 6:**
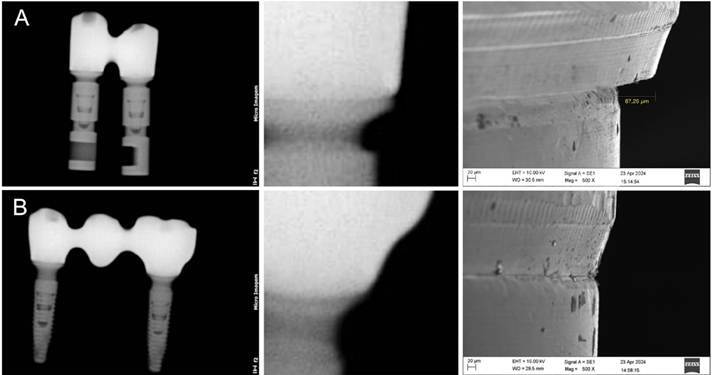
Radiographic and scanning electron microscopy (SEM) analysis. A: Example of a milled structure misfit on the distal face of the most posterior implant. B: Example of a milled structure without misfit.

A feasible correlation of misfit levels was identified in five radiographic images of the 15 milled structures screwed onto the multi-unit abutments of the implants. The misfit measurements visible in the radiographic images correlated with the respective measurements obtained through SEM analysis (Figure 6). Pearson's correlation revealed a strong positive relationship between the misfit values measured using SEM and those observed in the radiographs of the same samples (r = 0.819, *p = 0.045*).

## Discussion

The results of the present study demonstrated that varying inter-implant distances (7 mm, 14 mm, and 21 mm) did not significantly influence the trueness of virtual models or the marginal misfit of milled prosthetic structures. These findings contrast with previous reports, which indicate that longer inter-implant spans may reduce scanning accuracy due to accumulated stitching errors and increased complexity in data acquisition ^7^ ^,^ ^21^. The divergence in outcomes may be attributed to advancements in intraoral scanning systems, optimized scanning strategies, and improvements in the design and optical properties of scan bodies.

The trueness of the virtual models generated by intraoral scanning was not significantly affected by the varying distances between implants. This finding contrasts with previous studies that reported reduced precision at larger distances. ^2^ ^,^ ^8^ ^,^ ^9^ ^,^ ^10^ ^,^ ^11^ ^,^ ^13^ For example, a previous study concluded that intraoral scanners are prone to greater errors and lower reproducibility as inter-implant distances increase. ^7^ However, despite testing the exact distances as in the previously cited study, our results showed no statistically significant difference in the trueness of the virtual models or the misfit of the milled structures. Therefore, the null hypothesis was accepted.

To evaluate the accuracy of the intraoral scanner across the three models with varying inter-implant distances, reference data were generated using a high-precision desktop scanner (inEos X5, Dentsply Sirona). Trueness measurements were conducted by aligning the test models with the reference models and superimposing the data. Misalignment of the models indicates a translation error between the test objects and the reference objects. ^7^ ^,^ ^13^ The absence of significant differences in the means of the 3D comparisons between the reference and test groups suggests that the precision of the intraoral scanner remained consistent, regardless of the inter-implant distances. This discrepancy from earlier studies may be attributed to advancements in scanning protocols and technology, as well as the use of more refined scanning and alignment techniques in this study. Specifically, the high precision of the employed intraoral scanner likely contributed to the consistent accuracy of the virtual models, even with varying inter-implant distances. Furthermore, improvements in the design of scan bodies and their integration with the scanning software may have mitigated the impact of larger edentulous spaces.

The SEM examination revealed no vertical misfits, a significant finding, as vertical misfits generally pose greater concern than horizontal misfits due to their potential impact on the long-term stability and function of implant-supported restorations. ^21^ Although the precision of the digital models remained consistent, the increased misfit of milled structures, particularly at greater inter-implant distances, underscores the challenge of maintaining accurate prosthetic adaptation as implant spacing increases. ^2^ ^,^ ^8^ ^,^ ^10^ ^,^ ^12^ ^,^ ^22^ These findings suggest that despite advancements in scanning accuracy, further improvements are necessary in the manufacturing and fitting processes of prosthetic structures to address the clinical implications of larger inter-implant distances. This study highlights that digital workflows can maintain model accuracy across varying inter-implant distances while emphasizing the ongoing need for development in the adaptation of milled structures.

The radiographic analysis revealed that all misfits visible on radiographs corresponded to values ranging from 81.5 µm to 109.8 µm in the SEM analysis (mean: 89.5 µm, median: 86.2 µm). These values fall within a range considered clinically detrimental, as they can lead to complications in gingival and osseous tissues, ultimately compromising peri-implant health and implant survival. ^21^ ^,^ ^23^ These findings suggest that digital radiography is a reliable tool for assessing the adaptation of milled structures to implant abutments, offering valuable insights into the quality of implant-supported restorations. This radiographic approach provides a non-invasive and widely accessible method for evaluating prosthetic structure adaptation, complementing direct clinical measures and enabling a more comprehensive assessment of implant-supported restoration integrity. In this study, radiographic detection of misfit was possible for discrepancies as small as 81.5 µm, indicating that digital radiography can reliably identify clinically relevant misfits at this threshold. However, further studies, particularly clinical trials, are needed to validate this method as a dependable diagnostic tool for detecting misfits in implant-supported restorations.

Our findings suggest that intraoral scanning is a reliable method for capturing implant positions in short to moderate edentulous spans, potentially reducing the need for conventional impressions. Moreover, the strong positive correlation between misfit values detected by SEM and those observed in digital radiographs supports the feasibility of radiographic assessment as a non-invasive tool for evaluating prosthetic fit in clinical settings. ^5^

This *in vitro* study had several limitations. First, a typodont was used to simulate clinical conditions, which do not fully replicate the complexities of the oral cavity and natural teeth of real patients. In clinical settings, various factors can influence intraoral scanning results that are not present in an *in vitro* environment. For example, patient movement, limited mouth opening, the presence of the tongue, and hypersalivation can significantly impact the accuracy of intraoral scans. ^7^ ^,^ ^15^ Furthermore, radiographs were obtained without adjacent teeth, simplifying the assessment of misfits. In clinical scenarios, adjacent teeth can influence the seating and positioning of prostheses, potentially resulting in varied outcomes in terms of fit and adaptation. This study was purposefully designed to isolate the variable of inter-implant distance while maintaining all other parameters-such as scanning technology, scan body design, and operator technique-constant. This level of methodological control was essential to reduce confounding factors and to ensure that any observed differences could be confidently attributed to the spacing between implants. While this strategy enhanced the internal validity of our findings, we fully acknowledge that it may limit external generalizability, particularly across different intraoral scanning systems. To preserve consistency, a single intraoral scanner was employed throughout the study. This decision minimized potential variations stemming from equipment differences, allowing for a focused evaluation of inter-implant spacing. We acknowledge that the use of Ti-base components in clinical settings may not contribute to significant differences in marginal fit after preparation.

Furthermore, the type and geometry of the scan abutment itself may influence the accuracy of digital impressions by interacting with scanner-specific software algorithms. While our study controlled these variables to isolate the effect of inter-implant distance, we recognize that additional factors-such as different abutment materials and configurations (including Ti-base and castable options), scanner type, scan body material, and operator variability-can significantly influence digital impression accuracy and prosthetic adaptation. These elements should be thoroughly examined in future investigations to improve the reliability and clinical applicability of digital workflows.

Therefore, although this study provides valuable insights, its findings should be interpreted with caution, considering the inherent differences between *in vitro* and *in vivo* conditions, as well as the additional clinical factors that can influence intraoral scanning and prosthetic fit.

## Conclusion

Considering the limitations of this study, the findings indicate that the virtual models were accurate across all tested distances, and the milled structures adapted well to the implant abutments. Digital radiography proved to be a reliable method for analyzing structural adaptations.
